# Report of Surgical Cases*Read before Medical Association of Georgia, Athens, April, 1910.

**Published:** 1910-05

**Authors:** R. R. Kime

**Affiliations:** Atlanta, Ga.


### REPORT OF SURGICAL CASES.*

BY R. R. KIME, M. D., ATLANTA, GA.

Case 1.—Removal (3 gal.) ovarian cyst. Patient age 80 years. years.

Case 2.—Removal Septic Kidney. Patient age 62 years.

Case 3.—Removal (½ gal.) urine from right side. Patient age 11 years.

Case 4.—Vaginal Cæsarean section in Primipara for Eclampsia.

Case 5.—Operation Ectopic Pregnancy and Appendicitis.

Case 1.—Mrs. H., age 80 years, mother 12 children, youngest 32 years old, passed menopause without difficulty at 50, no miscarriages and no history of uterine or pelvic disease until present trouble.

First noticed enlargement June, 1908, nine months previous to operation. Had bloody vaginal discharge almost continuously for five months, but no evidence of malignant disease present. Inability to control urine at times; no swelling of extremities, senile changes in blood vessels affecting heart to some extent, urine contained hyaline and granular casts, round epithelim but no albumen.

Patient passed an unusual amount of urine involuntarily for

*Read before Medical Association of Georgia, Athens, April, 1910.

months and suffered pain and inconvenience from weight of tumor, so much so, that she requested removal of tumor if she died on operating table.

The patient was of slender build, tumor diagnosed as multilocular cyst, there being three distinct compartments in different degrees of tension, the largest with lowest tension on left side extending beyond median line of abdomen; the second with medium tension on right side of abdomen; the third on highest tension in right inguirnal region and not movable, all together containing about 3 gallons of fluid.

Two weeks of treatment, good nourishing diet, tonics, alimentary disinfectants and diuretics materially improved patient's general condition and she was operated on at Tabernacle Infirmary, March 6th, 1909.

Median incision, fluid evacuated slowly from two larger cavities so as not to remove abdominal pressure too rapidly and cause collapse of patient; broad pedicle tied off with chromic cat-gut, incision closed with layer cat-gut and Murphy fig. 8—silk worm gut sutures.

Hypodermoclysis used immediately after operation.

Patient passed an unusual amount of urine involuntary for several days after operation, otherwise made uneventful recovery, temperature not above 100 F. at any time, up in two weeks, left infirmary in three weeks after operation.

Tumor was not removed intact as advised by Mayos and Richardson (4 per cent. being malignant and liable to infection) because of the danger in this case from too sudden removal of intra-abdominal pressure producing callapse.

Besides the three large cavities mentioned, there were numerous smaller cysts, in each the fluid varid in color from clear-like water to straw, cherry, and almost black.

Patient gained rapidly after operation, continued tonic, diuretics and diet. Wrote May 12, 1909, that she was in better health than in years. Nine months later, patient returned to the city complaining of chills and fever, with pain, tenderness and some enlargement in right side.

Examination revealed enlargement size double fist, cystic, in right inguinal region, fluctuating, not movable and tender on pressure. Nothing felt by vaginal examination.

Being so long after operation, the cyst having been removed from the right side and tied off in pelvic cavity with cat-gut, we excluded infection from previous operation or from the ligatures.

Temperature ranged from 102° to 103° F., pulse 110 to 120, with evidences of septic infection which we thought was connected with rt. kidney. While investigating case, the tumor discharged its contents through the bladder, purulent, very offensive pus, temperature and pulse rapidly subsided, septic symptoms disappeared.

In three or four days a sudden, severe cutting pain came up, being summoned, but detained a few minutes with another patient, when I arrived the patient was easy, talking and laughing as if nothing had happened and showed me this largest stone about size and shape of 32 cartridge.

In a couple of days the same experience presented us with this second and smaller stone. After this the patient improved rapidly, urine cleared up materially—fewer casts, lucocytes, round epithe, etc.

The enlargement and tenderness in right side disappeared after the discharge through bladder and patient returned home a week after passing last calculus.

Patient being in Atlanta on a visit 1st of March of this year, had La Grippe, at which time I saw her twice. She soon recovered from acute attack, no evidence of former illness found.

Urine examined March, 1910: Simple Indican, pus cells, triple phosphates.

Case 2.—Mrs. G., widow, age 62 years, mother two children, youngest 28 years, suffering with hemorrhoids for which she was being prepared for operation when she came to Atlanta.

Found her with slight elevation of pulse and temperature, enlargement in right side, not very movable, somewhat tender, reaching from lower border of liver to right inguinal region, no history of renal, or hepatic colic, never had jaundice, no severe sickness, some mental derangement and some loss of flesh—history mostly negative.

Urine contained pus, small amount albumen. Round Ep.

granular and hyaline casts. Patient was sent to Tabernacle Infirmary for further investigation.

In two days temperature suddenly rose to 103° F., gradually dropping lower each day until 100° F., then another sudden rise without any distinct chill.

A surgeon in consultation diagnosed septic, distended gall bladder and advised operation accordingly.

Segregation of urine showed that from Lt. Kidney, had few lucocytes, some Rd. Ep. few gran. and hy. casts. Right kidney ʒi pus to ℥1 of urine, Rd. Ep., gran. and hyal. casts in abundance.

From urinary findings and careful palpation of tumor, I was more fully convinced my first diagnosis of diseased kidney was correct.

Patient growing rapidly worse, delirious at times, operation was consented to as being the only hope of relief.

Operation Dec. 21, 1907, incision one inch below twelfth rib in right lumbar region, kidney exposed, prolapsed and three times normal size, delivered and incised in Hyrtl's Exsanguinated Zone for examination, found numerous small pus foci and bled so freely closed up incision in kidney to check bleeding and did nephrectomy. Recognizing it as infectious, gauze and tubular drainage were elft in lumbas incision.

Kidney substance contained numerous pus foci and pus in pelvis. Pathologist reported streptococcic infection.

Patient recovered, mental condition materially improved and left kidney assumed function of right kidney without serious difficulty. Specimen of urine examined April 11, 1910. Did not show any casts but some lococytis probably mostly from vagnia.

This case was of special interest and reported on account of the difficulty in differentiating from septic disease of gall bladder.

Case 3.—F. A., girl, age 11 years, fell six weeks previous on sleeper of floor in a new building, landing on right side. Shortly afterward had what was classed as typhoid fever lasting three weeks, then clear of fever about two weeks.

At intervals complained of pain and some tenderness in right side.

On Saturday, July 11, 1908, patient had convulsions, elevation of pulse and temperature and enlargement in rightt tside first noticed. I saw patient next A. M., in semi-conscious condition, temperature about 101° F., pulse 115 to 120, a large fluctuation mass in right side reaching from brim of pelvis to one inch beyond imbillicus in median line, dullness continuous with liver.

With history and condition of patient, having passed some blood in urine soon after fall, injury of kidney, accumulation urine with commencing infection was diagnosed and operation advised.

Patient was moved same day to Atlanta, a distance of fifty miles, and operated on next A. M.

Incision one inch below and parallel to the twelfth rib on right side.

Four pints straw-colored urine followed with some blood and pus escaped through incision, kidney adherent could not be brought out without too much traumatism, palpated with finger, no perceptible rent in kidney substance or pelvis found, a roughened surface and notch in lower border of liver was felt.

Gauze and rubber drainage tube inserted, wound partially closed. Drainage kept up for three or four weeks. Urine discharged freely first week or two, then gradually lessened, and fistula completely closed in seven weeks.

Urine both before and after operation contained pus, round epithelium, gran. hyal. and epithelial casts, urine ultimately cleared up entirely and patient in good health.

Case 4.—Female, colored, age 18. Primipara, supposed to be seven and one-half months pregnant, taken with convulsions early A. M., elevation of pulse and temperature. I saw patient in convulsion at 10 A. M., having had several convulsions recurring every few minutes, unconscious, unable to swallow.

Hypodermics of Ver. Ver. Nor. also hyoscine and morphine were given. No labor pains, os small and not dilatable, almost complete suppression of urine.

Condition of patient demanded urgent action, vaginal cæsarean section was decided upon and patient moved to Spellman Hospital.

Operation P. M. of May 11, 1909. Transverse cervicovesical incision, bladder separated well up anterior wall of uterus cervix incised anteriorly and forceps applied.

Required considerable force to deliver foetus, was dead and probably a full term development could not be resuscitated.

Incisions closed with cat-gut sutures, patient made rapid recovery, kidney lesion cleared up, albumen, pus, gran. and hyaline casts abundant at first.

At Chicago meeting of A. M. A., 1908, in the obstetrical section, the leading speakers advised every country practitioner to be prepared to perform vaginal cæsarean section for puerperal eclampsia, that immediate delivery was demanded in all cases, and if parts are not in condition for such delivery, vaginal cæsarean section should be performed by the country practitioner.

Such advice is fraught with serious danger, and if followed, would add materially to the death rate in handling such cases.

It is a capital operation, and should only be done by one accustomed to surgical work.

There are very few cases that can not be more safely and better handled by other measures if the physician is properly qualified and understands obstetrical work.

A few hours' delay in delivery does not mean death in the great majority of cases, if the patient is properly handled and prepared for delivery. Yet there are a few cases in which vaginal cæsarean section is indicated and should be done.

*Case V.*—Mrs. T., age 28, married 3 years, flows regular since marriage, missed one week first of April, then appeared with pain and free flow, bowels obstinate to move, vomited food eaten one or two days previous, later passed undigested food by bowels. Two years previous had right sulpingitis, appendicitis, and local peritonitis which cleared up and operation declined.

This attack was supposed to be a rekindling of former trouble, bowels tender, swollen and painful, no satisfactory pelvic examination could be made.

Pain ceased, tenderness subsided, scant flow continued.

Two weeks later a second attack of pain, increased flow, vomiting, obstirpation, bowels tender, swollen and with thick abdominal walls precluded satisfactory examination, a provisional diagnosis of ectopic pregnancy was made and operation advised.

Operation April 20, 1909, just one year ago yesterday, which prevented my attending last annual meeting of this society, opening first made in vaginal vault to confirm diagnosis, then median incision in abdomen, omentum adherent to tubes, ovaries, uterus and intestines all in one mass, a portion of omentum was tied of dissected up and removed. Pelvis filled with blood and inflamatory exudate, intestines separated and found pregnancy had occurred in left tube and ruptured. No foetus found.

Tube and ovary so degenerated and brittle could not tie off, had to remove piece-meal and close raw surface and stop bleeding with continuous over and over cat-gut suture.

The extensive raw surfaces on intestines, uterus and in Douglas' Pouch could not be covered in, vaginal and abdominal drainage used.

The extreme critical condition of patient precluded prolonging operation to remove right tube, ovary and appendix.

Patient was placed in Fowler position and Proctoclysis used for nearly three days. Hypodermoclysis used at operation.

Patient made good recovery without hernia, although infection was present and affected abdominal incision.

Six months later patient had a rekindling of appendicitis, and trouble with right tube.

A second operation was performed Oct. 16, 1909, median incision dissecting out old scar, intestines adherent to right tube, uterus and old sight of ectopic pregnancy, appendix adherent to right tube and ovary.

Adhesion broken up right tube, ovary and appendix removed.

The tube as you see from specimen is more than one inch in diameter at fimbriated extremity and filled with a clear fluid which was probably purulent at first, having undergone a change from its primal development before removal.

Patient made an uneventful recovery, proctolysis being used as in former operation.

In closing this report, I desire to stress the importance of drainage, Fowler position and proctoclysis in critical cases of abdominal section with or without infection.

As ordinarily used proctoclysis is not of much benefit and very unsatisfactory, even in hospital work.

To obviate this difficulty and get the full benefit of the Murphy method, I have devised this apparatus which is simple, easily regulated and solution easily kept warm.

The apparatus can be used equally well in private as in hospital work and by any nurse of ordinary intelligence.

